# Involvement of the *N*-methyl-d-aspartate receptor GluN2D subunit in phencyclidine-induced motor impairment, gene expression, and increased Fos immunoreactivity

**DOI:** 10.1186/1756-6606-6-56

**Published:** 2013-12-16

**Authors:** Hideko Yamamoto, Etsuko Kamegaya, Wakako Sawada, Ryota Hasegawa, Toshifumi Yamamoto, Yoko Hagino, Yukio Takamatsu, Kazuhide Imai, Hisashi Koga, Masayoshi Mishina, Kazutaka Ikeda

**Affiliations:** 1 Addictive Substance Project, Tokyo Metropolitan Institute of Medical Science, 2-1-6 Kamikitazawa, Setagaya-ku, Tokyo 156-8506, Japan; 2 Laboratory of Molecular Psychopharmacology, Graduate School of Nanosciences, Yokohama City University, Yokohama, Kanagawa 236-0027, Japan; 3 Kazusa DNA Research Institute, Kisarazu, Chiba 292-0818, Japan; 4 Ritsumeikan University Research Organization of Science and Technology, Kusatsu, Shiga 525-8577, Japan

**Keywords:** GluN2C, GluN2D, Motor impairment, Motor loop, PCP

## Abstract

**Background:**

Noncompetitive *N*-methyl-d-aspartate (NMDA) receptor antagonists evoke a behavioral and neurobiological syndrome in experimental animals. We previously reported that phencyclidine (PCP), an NMDA receptor antagonist, increased locomotor activity in wildtype (WT) mice but not GluN2D subunit knockout mice. Thus, the aim of the present study was to determine whether the GluN2D subunit is involved in PCP-induced motor impairment.

**Results:**

PCP or UBP141 (a GluN2D antagonist) induced potent motor impairment in WT mice but not GluN2D KO mice. By contrast, CIQ, a GluN2C/2D potentiator, induced severe motor impairment in GluN2D KO mice but not WT mice, suggesting that the GluN2D subunit plays an essential role in the effects of PCP and UBP141, and an appropriate balance between GluN2C and GluN2D subunits might be needed for appropriate motor performance. The level of the GluN2D subunit in the mature mouse brain is very low and restricted. GluN2D subunits exist in brainstem structures, the globus pallidus, thalamus, and subthalamic nucleus. We found that the expression of the c-*fos* gene increased the most among PCP-dependent differentially expressed genes between WT and GluN2D KO mice, and the number of Fos-positive cells increased after PCP administration in the basal ganglia motor circuit in WT mice but not GluN2D KO mice.

**Conclusion:**

These results suggest that the GluN2D subunit within the motor circuitry is a key subunit for PCP-induced motor impairment, which requires an intricate balance between GluN2C- and GluN2D-mediated excitatory outputs.

## Background

The *N*-methyl-D-aspartate (NMDA) receptor channel is involved in various physiological functions, including learning and memory. NMDA receptors are primarily composed of GluN1, GluN2 (A-D), and GluN3 subunits. The GluN1 subunit is expressed ubiquitously, whereas GluN2 subunit expression is region- and cell-specific. Additionally, the temporal expression of each subunit varies [1]. Among the GluN2 (A-D) subunits, the physiological role of the GluN2D subunit is not fully understood because of its unique properties. In the rodent brain, the GluN2D subunit is expressed during postnatal periods. However, as the animal ages, the levels of GluN2D mRNA and protein decrease. In mature brains, significant levels of GluN2D subunits are mainly restricted to diencephalic, mesencephalic, and brainstem structures, particularly the globus pallidus, thalamus, subthalamic nucleus, and superior colliculus [1]. GluN2D knockout (KO) mice are viable and exhibit no obvious histological abnormalities [2]. However, these KO mice exhibit a reduction of spontaneous locomotor activity in a novel environment and less sensitivity to stress in the elevated plus maze, light–dark box, and forced swim test [2,3].

Phencyclidine (PCP) has been extensively used as a pharmacological model of schizophrenia because of its ability to evoke the positive and negative symptoms of schizophrenia and characteristic cognitive deficits of this illness observed in humans [4,5]. However, NMDA receptor antagonists evoke a behavioral syndrome in experimental animals characterized by hyperlocomotion and the disruption of prepulse inhibition of the startle response [6,7]. Chronic exposure to PCP intensely affects the relative immunoreactivity of the GluN2 subunit in the frontal cortex. Following PCP treatment, GluN2D immunoreactivity and protein expression increase significantly and induce a shift to a predominance of the GluN2D subunit in the frontal cortex [8]. However, the possible role of the GluN2D subunit in PCP-induced changes in gene expression and the brain networks involved in these changes remain unexplored.

We previously reported that the NMDA receptor antagonist PCP significantly increased extracellular levels of dopamine in the striatum and prefrontal cortex in wildtype (WT) mice but not GluN2D KO mice. Furthermore, acute and repeated administration of PCP did not increase locomotor activity in GluN2D KO mice [9].

In the present study, we performed the rotarod test, gene expression analyses, and Fos immunohistochemistry in WT and GluN2D KO mice to elucidate the role of the GluN2D subunit in PCP-induced behavioral and neurobiological effects. The results indicated that the GluN2D subunit of the NMDA receptor plays an important role in PCP-induced motor impairment and PCP-induced abnormal gene expression. PCP-induced motor impairment was attenuated in GluN2D KO mice by preventing PCP-induced excitation via the GluN2D subunit in the neural circuit that includes the motor loop.

## Results

### Pharmacological modulation of motor performance using PCP and UBP141

**Figure 1:**
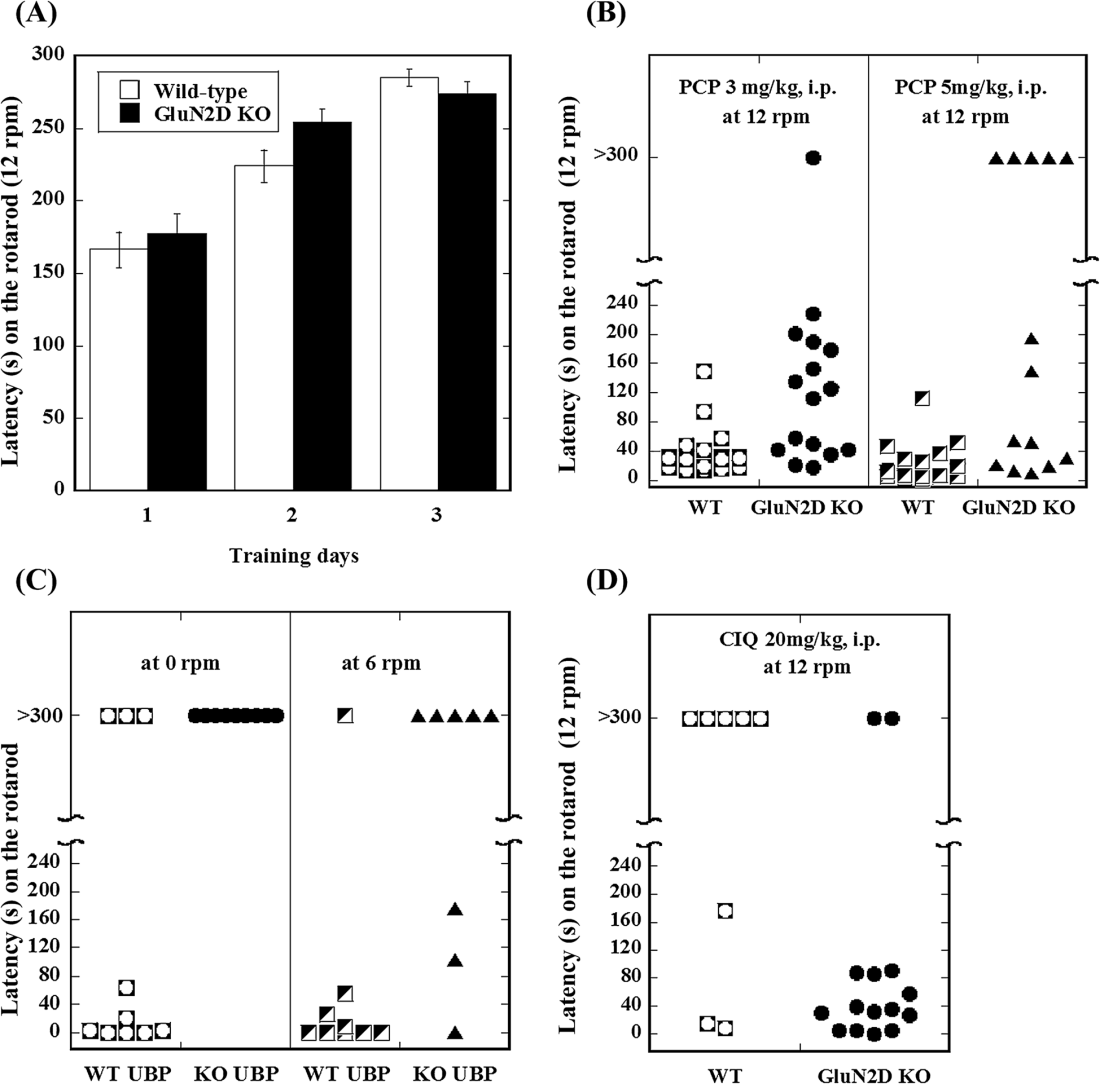
**The effects of PCP, UBP, and CIQ on motor performance. (A)** Wildtype mice and KO mice did not display significant differences in motor learning. WT, wildtype mice (*n* = 60); KO, GluN2D knockout mice (*n* = 55). **(B)** Ataxic effects of phencyclidine (PCP) at 3.0 mg/kg (*n* = 15 WT, *n* = 17 KO) and 5.0 mg/kg (*n* = 16 WT, *n* = 15 KO) in the rotarod test at 12 rpm. A significant effect of genotype was observed in the PCP-induced decrease in time at the two doses tested (*p* < 0.05 at both doses, dependence on genotype; Fisher’s exact test). Twenty-four to 72 h after PCP injection, the WT and KO mice were injected with saline. Motor performance was not affected by saline injection. Almost all of the mice showed maximal performance at 12 rpm (> 300 s), and the remaining mice showed normal performance (> 200 s). **(C)** Effect of UBP in the rotarod test at 0 rpm (*n* = 10 WT, *n* = 9 KO) and 6 rpm (*n* = 9 WT, *n* = 8 KO). The differences between WT and GluN2D KO mice in UBP141-induced motor impairment were significant at 0 and 6 rpm (*p* < 0.05 at both speeds, dependence on genotype; Fisher’s exact test). The vehicle injection did not affect motor performances in WT and KO mice (both genotypes: > 300 s at 12 rpm, *n* = 5; > 200 s at 12 rpm, *n* = 2; < 200 s at 12 rpm, *n* = 1). **(D)** Effect of CIQ in the rotarod test at 12 rpm (*n* = 8 WT, *n* = 15 KO). The difference between WT and GluN2D KO mice in CIQ-induced motor impairment was significant (*p* < 0.05, dependence on genotype; Fisher’s exact test). The vehicle injection did not affect motor performances in WT and KO mice (WT: > 300 s at 12 rpm, *n* = 5; > 200 s at 12 rpm, *n* = 2; KO: > 300 s at 12 rpm, *n* = 11; > 200 s at 12 rpm, *n* = 2).

Motor impairment was measured by placing WT and GluN2D KO mice on a fixed-speed rotarod (12 rpm) and monitoring their performance for 300 s, 30 min after saline or PCP administration. Saline injections did not affect motor performances. At a dose of 3 or 5 mg/kg, PCP led to motor impairment in the rotarod test (Figure 1B). PCP-injected GluN2D KO mice performed better than PCP-injected WT mice. Significant differences in motor impairment were observed between WT and GluN2D KO mice, based on Fisher’s exact test (*p* < 0.05 at both doses; dependence on genotype).

The UBP141 solution (3 mM) was freshly prepared and injected intracisternally under ether anesthesia. Motor impairment was measured by placing WT and GluN2D KO mice on a fixed-speed rotarod (0 rpm and 6 rpm) and monitoring their performance for 300 s, 30 min after UBP141 administration. Motor performance was maintained after vehicle injections. An intracisternal injection of 3 mM UBP141 solution (20 μl) resulted in motor impairment in seven of 10 WT mice at 0 rpm and eight of 9 WT mice at 6 rpm (Figure 1C). In contrast, UBP141 did not cause motor impairment in GluN2D KO mice at 0 rpm (*n* = 9) and five of eight KO mice at 6 rpm (Figure 1C). The differences in UBP141-induced motor impairment between WT and GluN2D KO mice were significant at 0 rpm and 6 rpm (Fisher’s exact test, *p* = 0.0031 and 0.049, respectively; dependence on genotype).

The CIQ solution and its vehicle were freshly prepared and administered in WT and GluN2D KO mice. CIQ did not cause hyperlocomotion, but it markedly reduced motor performance in 13 of 15 GluN2D KO mice (Figure 1D). In contrast, CIQ did not cause motor impairment in five of eight WT mice. The difference in CIQ-induced motor impairment between WT and GluN2D KO mice was significant (Fisher’s exact test, *p* = 0.0026; dependence on genotype).

### Altered gene expression in GluN2D KO mice in response to PCP

**Figure 2:**
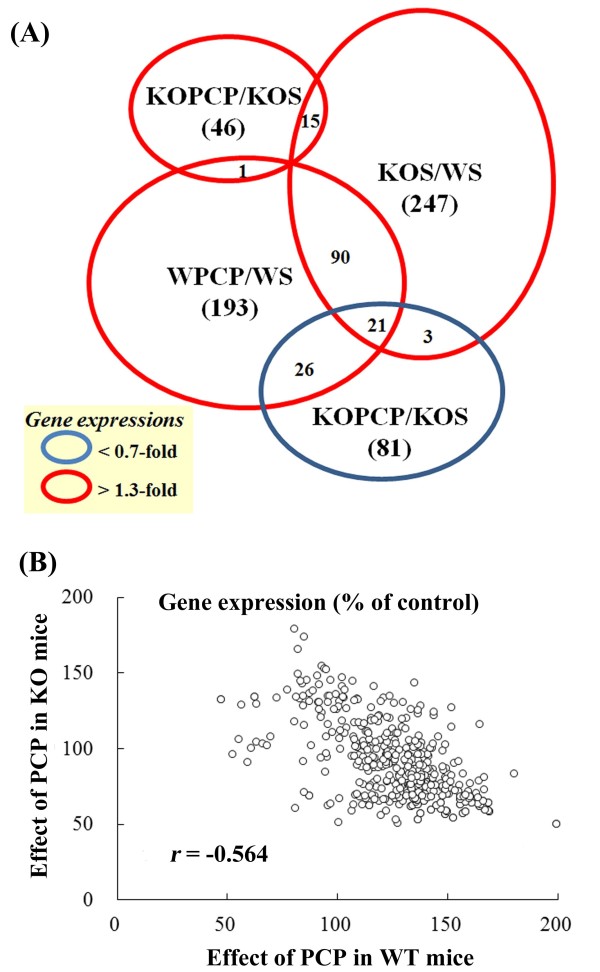
**The preliminary cDNA array experiment. (A)** Venn diagrams that illustrate the distribution of altered transcripts in the striatum. The diagrams are derived from the gene expression data of the cDNA array data (preliminary experiment). The red circle or blue circle contains the genes that exhibited increased or decreased expressions more than 1.3-fold or less than 0.7-fold compared with saline-injected control mice, respectively. The numbers in parentheses show the number of changed transcripts in each group. WPCP, PCP-injected WT mice; WS, saline-injected WT mice; KOS, saline-injected GluN2D KO mice; KOPCP, PCP-injected GluN2D KO mice. **(B)** Scatter plot of the effect of PCP on gene expression in WT mice (X-axis) and GluN2D KO mice (Y-axis). The scatter plot of all of the transcripts shows the ratios between the expression of each gene in WPCP and the expression of each gene in WS and the ratios between the expression of each gene in KOPCP and the expression of each gene in KOS. Each point shows the fold-change (%) using the cDNA array data (preliminary experiment), which included globally normalized data. The associations between the parameters were analyzed using Karl Pearson’s correlation or Spearman’s rank correlation. Gene expression in WPCP was negatively correlated with gene expression in KOPCP (*r* = −0.564, *p* < 0.01).

We explored the alterations in gene expression in GluN2D KO mice that could be responsible for the differential behavioral responses to PCP. We first performed a preliminary cDNA array experiment using mRNA derived from the striata in WT and GluN2D KO mice with or without PCP treatment. Compared with saline-injected WT mice, saline-injected GluN2D KO mice showed increased expression of 247 transcripts (Figure 2A; KOS/WS). An injection of PCP (10 mg/kg, s.c.) in WT mice and KO mice increased the expression of 193 and 46 transcripts, respectively (Figure 2A; WPCP/WS or KOPCP/KOS). The expression of a large number of genes was increased in the saline-injected GluN2D KO and PCP-injected WT mice. Both groups shared 36% and 46% of the increased transcripts, respectively. However, the increased gene expression pattern in PCP-injected GluN2D KO mice (KOPCP/KOS) differed from PCP-injected WT mice (WPCP/WS). Interestingly, 47 and 24 of the transcripts that showed increased expression in PCP-injected WT mice (WPCP/WS) and saline-injected GluN2D KO mice (KOS/WS), respectively, showed an opposite trend in PCP-injected GluN2D KO mice (Figure 2A). Figure 2B illustrates the relationship between the effect of PCP in WT and KO mice. Each control consisted of a WT or KO mouse. Linear regression analysis showed that the data fit a straight line (*r* = −0.564). The expression ratios of all of the transcripts demonstrated a weak inverse relationship in response to PCP between WT and KO mice. The probes in this cDNA array contain many interesting genes but do not fully cover genes. Therefore, we next used a commercial array (MouseRef-8 BeadChip with greater than 24,000 probes) provided by Illumina.

We utilized WT and GluN2D KO mouse striata to profile the DEGs following PCP administration in WT and GluN2D KO mice. Total RNAs from the striata of WT and GluN2D KO mice obtained 2 h after an injection of saline or PCP (10 mg/kg, s.c.) were analyzed using a microarray-based screen with Illumina MouseRef-8. An Additional file 1: Table S1 shows this in more detail [see Additional file 1: Table S1]. This comparative analysis revealed the differential expression profiles of 51 genes following PCP administration, with 40 genes upregulated more than 1.3-fold and 11 genes downregulated less than 0.7-fold in WT and GluN2D KO mice. The two genotypes were found to share only 18% of the genes in the PCP-induced increase in the expression profile. Correlation analysis revealed that the PCP-induced increase in gene expression in WT and GluN2D KO mice was inversely associated (*r* = −0.46).

### Real-time qRT-PCR analyses

To validate the microarray data, the RNA levels of some of the candidate DEGs were analyzed by qRT-PCR. From the DNA array results using the striata, 23 genes that were upregulated more than 1.3-fold or downregulated less than 0.7-fold in WT and GluN2D KO mice were chosen. The qRT-PCR results (Table 1) confirmed the microarray data for both the upregulation and downregulation of the selected genes. The expression of *Hace1*, *Osbpl8*, *Smpd4*, and *Slc17* in the striatum in GluN2D KO mice was significantly increased. Among the 23 genes, 17 were upregulated, and one was downregulated with PCP treatment. Several of these 17 genes in GluN2D KO mice showed significantly altered expression in response to PCP compared with their expression profiles in WT mice.

**Table 1:** qRT-PCR validation of genes selected from fold change analysis of microarray data in the striatum

**Symbol**	**Definition**	***n***	**KOS/WS (vs. WS) %**	**WPCP/WS (vs. WS) %**	**KOPCP/KOS %**	**(vs. WPCP/WS)**
***Significantly changed gene expressions of NR2D KO mice***						
**Slc17**	*vesicular glutamate transporter 2*	4	210 ± 39 *	117 ± 14	49 ± 12	c
**Hace1**	*HECT domain and ankyrin repeat containing, E3 ubiquitin protein ligase 1*	7	157 ± 12 **	101 ± 9	84 ± 5	a
**Smpd4**	*sphingomyelin phosphodiesterase 4* (FLJ20297)	6	147 ± 4 **	105 ± 7	86 ± 5	b
**Osbpl8**	*oxysterol binding protein-like 8* (ORP8)*, transcript variant 1*	4	136 ± 8 **	112 ± 10	68 ± 7	c
***Significantly changed gene expressions induced by PCP of wild-type mice***						
**Fos**	*FBJ osteosarcoma oncogene*	4	110 ± 8	283 ± 40 *	109 ± 12	c
**Txnip**	*thioredoxin interacting protein (Txnip), transcript variant 1*	4	137 ± 5 *	211 ± 29 **	122 ± 11	b
**Pglyrp1**	*peptidoglycan recognition protein 1* (PGRP-S)	4	120 ± 5	176 ± 7 **	126 ± 6	c
**Fosb**	*FBJ osteosarcoma oncogene B*	3	122 ± 3	162 ± 29 **	74 ± 9	b
**Sesn1**	*sestrin 1*	4	116 ± 6	155 ± 11 **	120 ± 7	b
**Arrdc**	*arrestin domain containing 3 (mKIAA1376)*	4	133 ± 8 **	149 ± 10 **	95 ± 9	c
**Zbtb4**	*zinc finger and BTB domain containing 4*	4	115 ± 5	141 ± 3 *	109 ± 6	c
**Bag-3**	*Bcl2-associated athanogene 3*	4	107 ± 3	132 ± 5 **	108 ± 7	b
**Ddit4**	*DNA-damage-inducible transcript 4* (REDD1)	4	124 ± 10	184 ± 27 **	142 ± 19	a
**Gadd45g**	*growth arrest and DNA-damage-inducible 45* γ	4	119 ± 5	170 ± 15 *	126 ± 26	a
**Tsc22d3**	*TSC22 domain family, member 3, transcript variant 1* (DSIPI)	4	100 ± 4	154 ± 18 *	148 ± 17	a
**Cbr3**	*carbonyl reductase 3*	4	102 ± 5	152 ± 6 **	149 ± 17	a
**Cfp**	*complement factor properdin* (Properdine)	4	133 ± 34	142 ± 14 *	126 ± 34	a
**Sbf1**	*SET binding factor 1*	6	91 ± 10	141 ± 14 *	217 ± 65	a
**Slc2a1**	*solute carrier family 2* (GLUT1)	4	103 ± 8	140 ± 10 **	136 ± 14	a
**Sgk1**	*serum/glucocorticoid regulated kinase 1*	4	120 ± 11	138 ± 9 **	114 ± 16	a
**Rgc32**	*RIKEN cDNA 1190002H23 gene*	4	104 ± 4	133 ± 7 *	125 ± 12	a
**Egr1**	*early growth response 1(zif-268)*	4	114 ± 11	60 ± 5 *	57 ± 10	a
***Significantly changed gene expressions induced by PCP of GluN2D KO mice***						
**Egr4**	*early growth response 4*	4	121 ± 9	82 ± 10	48 ± 4	b

Data are expressed as the mean percentage ± SEM relative to the saline treated controls.

Compared to WS controls; *, *p* <0.05. **, *p* <0.01.

WPCP/WS vs. KOPCP/KOS; a, not significant. b, *p* <0.05. c, *p* <0.01.

Similarly, we evaluated total RNA derived from pooled frontal cortices in the qRT-PCR analyses (Table 2). Using the same primer sets that we used for the striatum, 13 genes were found to be upregulated by PCP treatment. As a result, we found four candidate genes from the cDNA array and confirmed their expression using real-time RT-PCR. These four genes are not involved in the MouseRef-8 BeadChip. Furthermore, we found 23 candidate genes from the MouseRef-8 BeadChip with greater than 24,000 probes.

**Table 2:** **qRT-PCR analysis of genes using the frontal cortex (the same genes in Table **1**)**

**Symbol**	**Definition**	***n***	**KOS/WS (vs. WS) %**	**WPCP/WS (vs. WS) %**	**KOPCP/KOS %**	**(vs. WPCP/WS)**
***Significantly changed gene expressions induced by PCP of wild-type mice***						
**Fos**	*FBJ osteosarcoma oncogene*	4	89 ± 7	271 ± 16 **	179 ± 5	c
**Fosb**	*FBJ osteosarcoma oncogene B (Fosb)*	3	98 ± 7	246 ± 69 **	134 ± 19	a
**Ddit4**	*DNA-damage-inducible transcript 4* (REDD1)	3	112 ± 5	200 ± 8 **	164 ± 4	b
**Egr4**	*early growth response 4*	4	107 ± 19	164 ± 56 **	86 ± 3	b
**Tsc22d3**	*TSC22 domain family, member 3, transcript variant 1 (DSIPI)*	4	100 ± 7	209 ± 24 *	190 ± 15	a
**Gadd45g**	*growth arrest and DNA-damage-inducible 45 γ*	4	103 ± 2	189 ± 25 *	146 ± 7	a
**Cfp**	*complement factor properdin* (Properdine)	4	105 ± 19	151 ± 9 **	126 ± 20	a
**Slc2a1**	*solute carrier family 2* (GLUT1)	4	93 ± 5	149 ± 8 **	150 ± 5	a
**Txnip**	*thioredoxin interacting protein, transcript variant 1*	4	106 ± 8	143 ± 16 **	126 ± 10	a
**Bag-3**	*Bcl2-associated athanogene 3*	4	91 ± 6	142 ± 6 **	128 ± 6	a
**Pglyrp1**	peptidoglycan recognition protein 1 (PGRP-S)	3	93 ± 5	139 ± 12 **	128 ± 12	a
**Arrdc**	*arrestin domain containing 3 (mKIAA1376)*	3	110 ± 1	136 ± 14 *	115 ± 14	a
**Rgc32**	*RIKEN cDNA 1190002H23 gene*	4	97 ± 6	131 ± 7 *	123 ± 6	a
***Significantly changed gene expressions induced by PCP of NR2D KO mice***						
**Egr1**	*early growth response 1(zif-268)*	4	94 ± 8	87 ± 8	67 ± 3	a

Data are expressed as the mean percentage ± SEM relative to the saline treated controls.

Compared to WS controls; *, *p* <0.05. **, *p* <0.01.

Compared to WS controls; *, *p* <0.05. **, *p* <0.01. WPCP vs. KOPCP; a, not significant. b, *p* <0.05. c, *p* <0.01.

### Bioinformatics analyses

**Figure 3:**
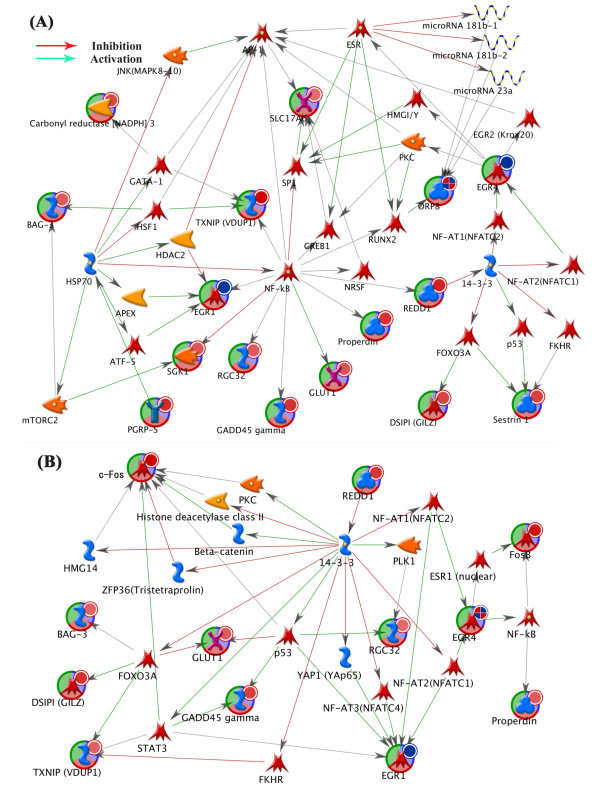
**Network developed in MetaCore based on the genes listed in (A) Table **1**and (B) Table **2**.** Interactions between nodes are shown by arrows, indicating activation (green), inhibition (red), and unspecified effects (gray). Detailed information on the symbols can be found at http://pathwaymaps.com/pdf/MC_legend.pdf (accessed July 7, 2013). A gene in the networks that contain a small circle was verified by quantitative reverse-transcription polymerase chain reaction. The small red circle indicates upregulation. The small blue circle indicates downregulation. The small mixed-color circle indicates positively and negatively regulated expression dependent on genotype.

The gene expression data shown in Tables 1 and 2 were uploaded into MetaCore 5.0 software (GeneGo pathway analysis). Graphical representations of the molecular relationships between genes were generated in a network built by the auto expand path algorithm, allowing 50 nodes (Figure 3). The network analysis of the 16 genes in Figure 3 showed that eight genes were directly linked to nuclear factor-κB (NF-κB; Figure 3A). Downstream of NF-κB was indirectly linked to 14-3-3 as one node and then from 14-3-3 spread to EGR4 or DSIPI and Sestrin 1. However, significantly altered processes for KOPCP/KOS were not indicated. Similarly, 12 genes in the frontal cortex (Table 2) generated a network (Figure 3B). This network included TXNIP (an oxidative stress mediator), REDD1 (which promotes neuronal cell death), and GADD45γ (which is involved in the regulation of growth and apoptosis). These analyses indicated that PCP is a stress factor that induces stress responses and is involved in apoptosis in WT mice. Among the PCP-induced changes in gene expressions, Fos was most distinguishable between WT and GluN2D KO mice. Therefore, we chose Fos for the immunohistochemical analysis.

### Induction of Fos by PCP administration

**Figure 4:**
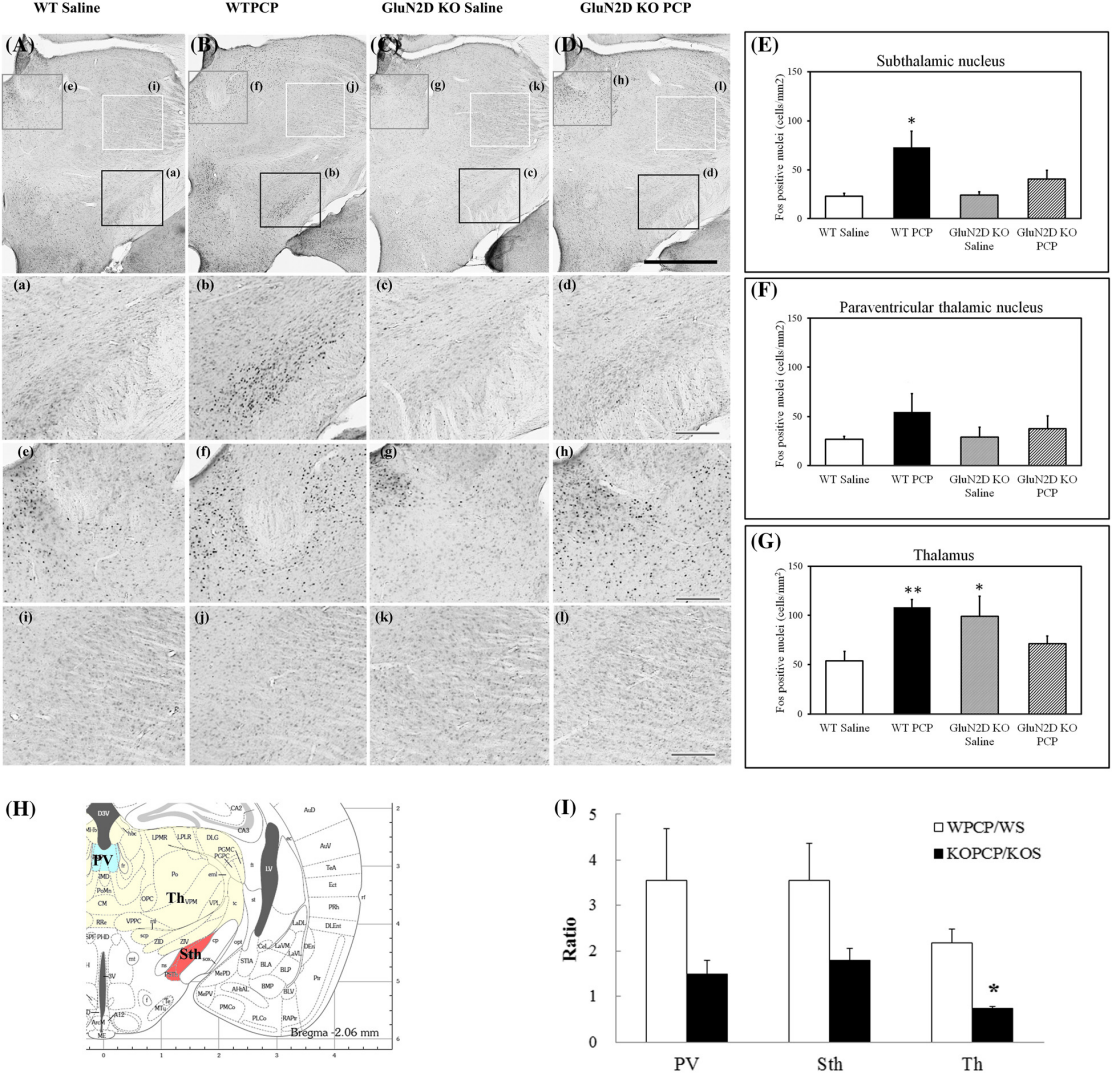
**Analyses of Fos induction in various brain regions in wildtype and GluN2D knockout mice.** The photomicrographs illustrate the immunohistochemical labeling of Fos in coronal sections of the thalamus. The boxes are shown in panels **a-i** are at higher magnification. A schematic representation of the paraventricular thalamic nucleus (PV), thalamus (Th), and subthalamic nucleus (Sth) is shown in panel **H**. The number of Fos-positive cells per square millimeter was determined in the respective brain areas **(E–G)** and normalized to the number in saline-treated mice **(I)**. Open bars, wildtype (WT) mice; closed bars, GluN2D KO mice. The results are expressed as the mean ± SEM ratio between the density of Fos-positive neurons in PCP-treated mice to the density of Fos-positive neurons in saline-injected mice (*n* = 4–6). One-way ANOVA of the PCP-induced increase in Fos-positive cells indicated a significant effect of genotype in the thalamus (Kruskal-Wallis nonparametric ANOVA followed by Scheffe’s test, *p* = 0.0209, unequal variance). *p < 0.05. Scale bars = 1 mm and 200 μm in panel **D** and **d**, respectively. The brain regions are defined as indicated by the images in the atlas [10].

**Figure 5:**
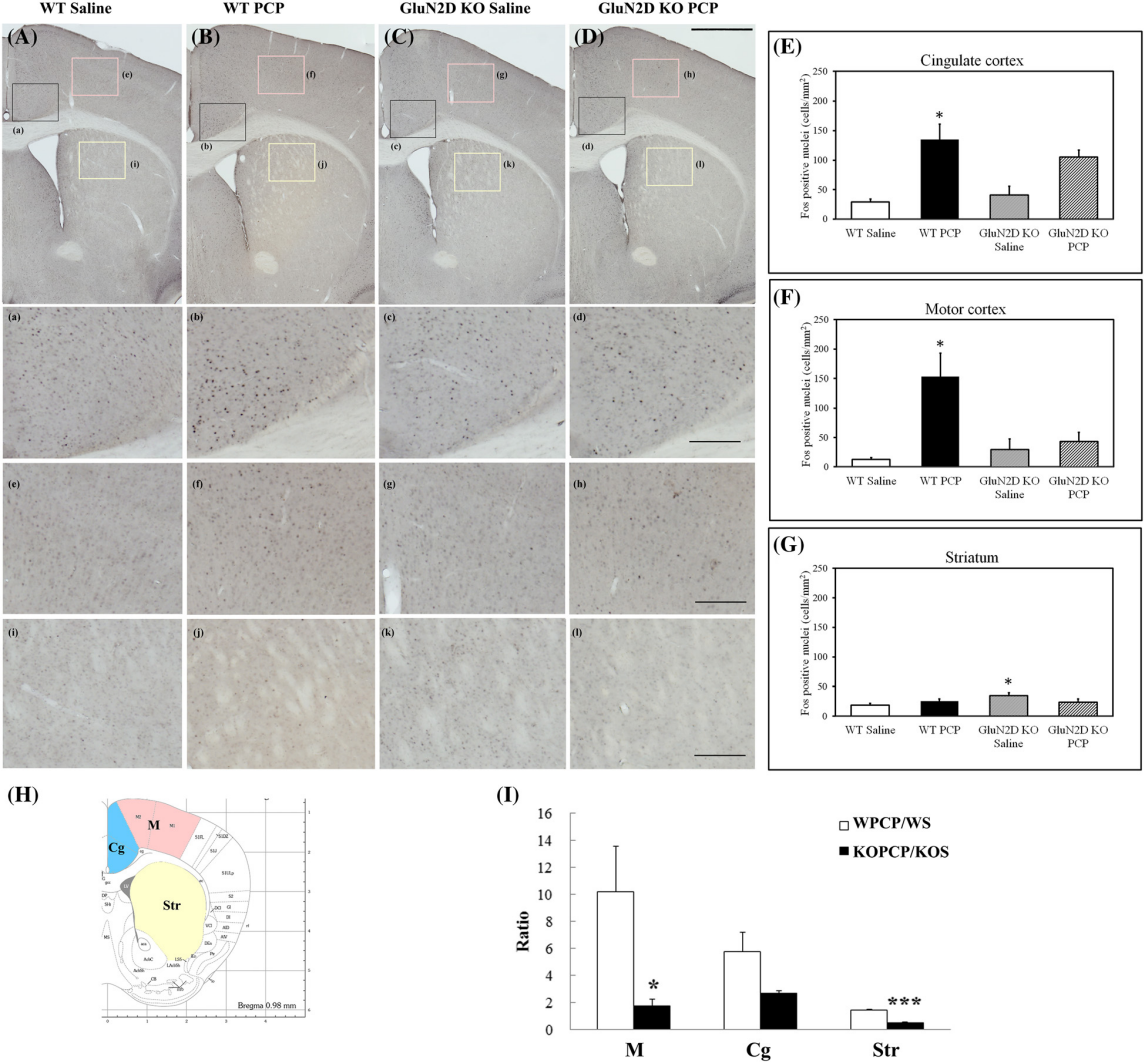
**Analyses of Fos induction in various brain regions in wildtype and GluN2D knockout mice.** The photomicrographs illustrate the immunohistochemical labeling of Fos in coronal sections of the forebrain. The boxes are shown in panels **a-i** are at higher magnification. A schematic representation of the cingulate cortex (Cg), motor cortex (M), and striatum (Str) is shown in panel **H**. The number of Fos-immunoreactive cells per square millimeter in PCP-treated mice was determined in the respective brain regions **(E–G)** and normalized to the number in saline-treated mice **(I)**. Open bars, wildtype (WT) mice; closed bars, GluN2D KO mice. The results are expressed as the mean ± SEM ratio between the density of Fos-positive neurons in PCP-treated mice to the density of Fos-positive neurons in saline-injected mice (*n* = 4–6). One-way ANOVA of the PCP-induced increase in Fos-positive cells indicated a significant effect of genotype in the striatum (*F*_1,6_ = 75.8, *p* = 0.0001). *p < 0.05, ***p < 0.001. Scale bars = 1 mm and 200 μm in panel **D** and **d**, respectively. The brain regions are defined as indicated by the images in the atlas [10].

Photomicrographs and semiquantitative analyses of Fos expression are shown in Figures 4 and 5. PCP (10 mg/kg, s.c.) markedly increased the density of Fos-positive cells in the motor cortex and cingulate cortex and moderately increased the density of Fos-positive cells in the subthalamic nucleus, and thalamus in WT mice (Figures 4EG and 5EF). However, the Fos ratio was scarcely increased in the striatum (Figure 5). Two-way ANOVA of the Fos data in the motor cortex indicated a significant effect of PCP treatment (effect of PCP, *F*_1,15_ = 10.59, *p* = 0.0053; PCP-genotype interaction, *F*_1,15_ = 7.18, *p* = 0.017). Further, post hoc testing by Scheffe’s test revealed that PCP significantly increased Fos density in the motor cortex of WT mice (*p* = 0.0006). In the cingulate cortex and subthalamic nucleus, two-way ANOVA indicated a significant effect of PCP treatment (cingulate cortex, *F*_1,16_ = 29.17, *p* = 0.0001; subthalamic nucleus, *F*_1,16_ = 11.44, *p* = 0.0038, respectively. no PCP-genotype interaction). Due to unequal variance, we used Kruskal-Wallis nonparametric test. It indicated that PCP significantly increased Fos density in the cingulate cortex (*p* = 0.024) and the subthalamic nucleus (*p* = 0.046) of WT mice. Additionally, two-way ANOVA of Fos-data in the thalamus indicated a significant PCP-genotype interaction (*F*_1,12_ = 10.74, *p* = 0.0066) and followed by Scheffe’s test, a significant increase in GluN2D KO mice treated with saline compared to WT mice (*p* = 0.0255) (Figure 4G). Two-way ANOVA of Fos-data in the striatum indicated no main effect and no PCP-genotype interaction and followed by Scheffe’s test, a significant increase in GluN2D KO mice treated with saline compared to WT mice (*p* = 0.0240) (Figure 5G). In the present study, the PCP-induced increase in the number of Fos-positive cells was suppressed to varying degrees in the observed brain areas in GluN2D KO mice.

### Induction of c-fos mRNA by UBP141, CIQ, and PCP with dantrolene administration determined by qRT-PCR

**Figure 6:**
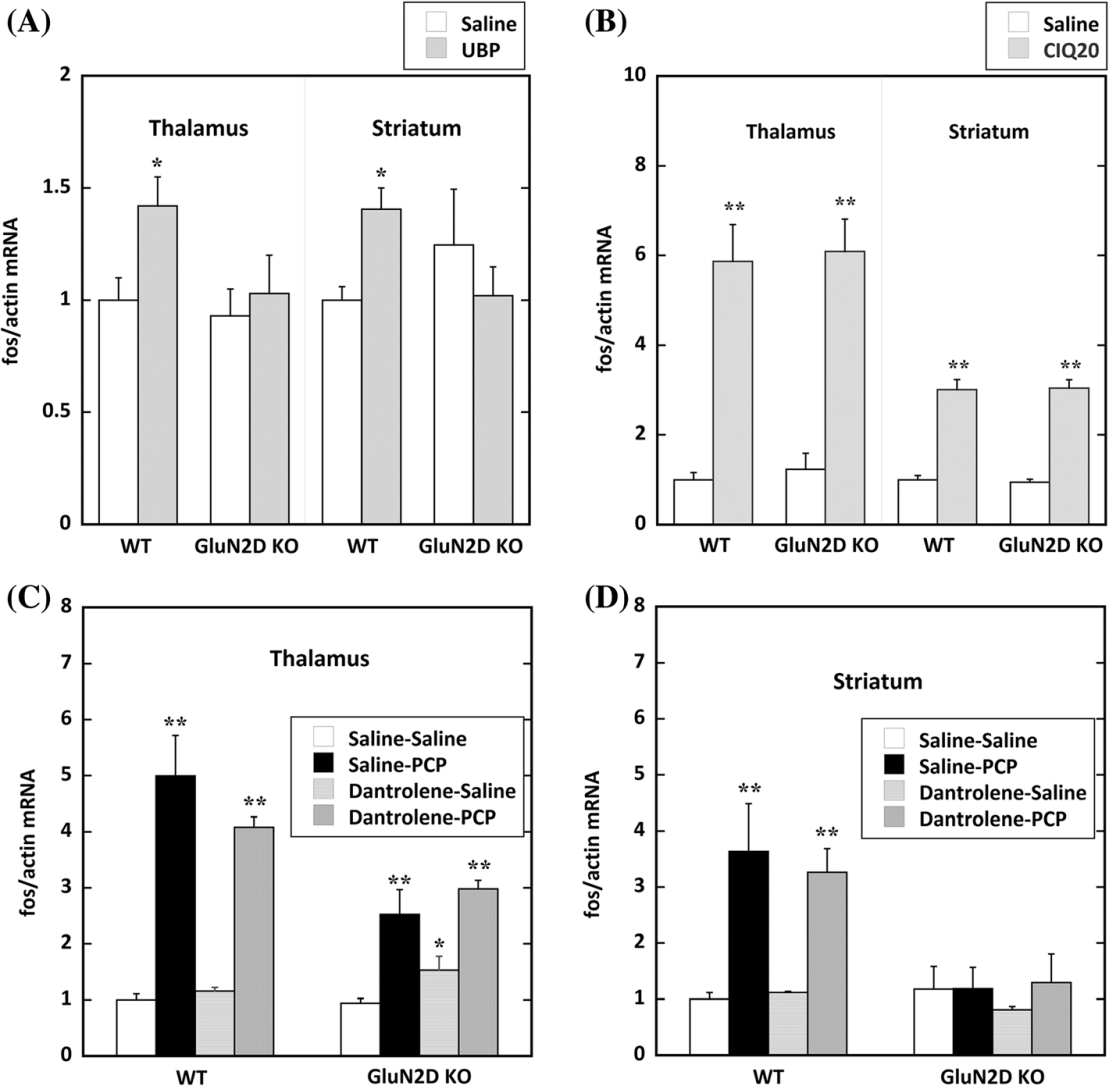
**Quantitative RT-PCR demonstrated the transcription of c-*fos *expression in the thalamus and striatum.** The ratio of c-*fos*/*actin* transcripts is expressed as the mean ± SEM fold change from saline controls in WT mice. **(A)** Two hours after the injection of UBP141 (3 mM, 20 μl, i.c.), a significant increase in c-*fos* mRNA was observed in the thalamus and striatum in WT but not KO mice (*p < 0.05). **(B)** CIQ (20 mg/kg, i.p.) markedly increased c-*fos* mRNA expression in the thalamus and striatum in WT and KO mice (**p < 0.01). No genotype difference was found between WT and KO mice. **(C)** PCP (10 mg/kg, s.c.) significantly increased c-*fos* mRNA expression in the thalamus with or without dantrolene (**p < 0.01), which completely suppressed PCP-induced hyperlocomotion. In the thalamus, dantrolene (5 mg/kg, i.p.) itself induced the slight activation of c-*fos* expression in GluN2D KO mice (*p < 0.05). **(D)** PCP (10 mg/kg, s.c.) significantly increased c-*fos* mRNA expression in the striatum in WT mice with or without dantrolene, but PCP had no effect in the striatum in GluN2D KO mice.

The levels of c-*fos* mRNA were examined by qRT-PCR in the striatum and thalamus in WT and GluN2D KO mice. Intracisternal injection of UBP141 enhanced c-*fos* expression in the thalamus and striatum in WT mice (*F*_1,11_ = 7.102, *p* = 0.016, and *F*_1,11_ = 4.585, *p* = 0.048, respectively) but not in GluN2D KO mice (Figure 6A). We then used CIQ, a potentiator of NR2C/2D subunits. CIQ markedly enhanced c-*fos* expression in the thalamus and striatum in both WT and GluN2D KO mice (Figure 6B). In the thalamus and striatum, two-way ANOVA indicated a significant effect of CIQ treatment (*F*_1,19_ = 112.59, *p* < 0.0001, and *F*_1,19_ = 197.01, *p* < 0.0001, respectively).

In the present study, we found substantial genotype-dependent differences in PCP-induced c-*fos* expression. This difference might have been secondary to PCP’s differential genotypic effects on hyperlocomotion. Therefore, we injected mice with dantrolene before PCP administration to investigate the effect of hyperlocomotion on c-*fos* expression stimulated by PCP. Dantrolene acts as muscle relaxant and is used for malignant hyperthermia. The therapeutic action of dantrolene appears to be attributable to its ability to bind to an amino-terminal sequence of skeletal ryanodine receptors [11]. Dantrolene completely blocked PCP-induced hyperlocomotion in WT mice (*n* =3). Dantrolene itself did not increase c-*fos* expression except in the thalamus in GluN2D KO mice (Figure 6C). Dantrolene did not inhibit PCP-induced c-*fos* expression in the thalamus or striatum in WT mice or thalamus in GluN2D KO mice (Figure 6C, D). Therefore, the substantial genotype differences in PCP-induced c-*fos* expression were not secondary to PCP’s differential genotypic effects on hyperlocomotion. In addition, Numeric data of Figure 6(A-D) and these actin data are shown in Additional file 2: Table S2.

## Discussion

The pharmacological activity of acute PCP administration in mice can be broadly classified into three symptoms: cognitive dysfunction, hyperlocomotion, and motor impairment. In the present study, we focused on motor impairment caused by PCP. The main findings were that PCP-induced motor impairment was markedly reduced in GluN2D KO mice compared with WT mice. The PCP-induced enhanced expression of c-*fos*, one of the most potent PCP-dependent DEGs between the two genotypes, was suppressed in several areas of the motor circuit in GluN2D KO mice. These findings indicate that ablation of the GluN2D subunit blocks the PCP-dependent abnormal neuronal activation in the motor circuit and prevents motor impairment. Thus, GluN2D subunit-containing NMDA receptors appear to play a key role in PCP-induced motor impairment.

### PCP-induced motor impairment is attributable to blockade of the GluN2D subunit

Although PCP is a nonselective blocker of GluN2(A-D)-containing NMDA receptors [12], PCP-induced motor impairment was reduced in GluN2D KO mice, suggesting that systemic PCP administration resulted in motor impairment mainly by blocking the GluN2D subunit. Watanabe *et al.* reported that the expression of GluN2D subunit mRNA is clearly detectable in the diencephalon and brainstem in the embryonic brain, but GluN2D subunit expression is nearly abolished in the mature rodent brain [1]. In the present study, the striatum had low expression levels of the GluN2D subunit. Interestingly, rather low levels of the GluN2D subunit play an important role in PCP-induced abnormal behaviors.

Ablation of the GluN2D subunit did not produce motor impairment in mature GluN2D KO mice, but change in gene expression remained. In the striatum of GluN2D KO mice, we found increased expression of four genes: *Slc17*, *Hace1*, *Smpd4*, and *Osbpl8*. Among these genes, the expression of *vGluT2* (*Slc17*), which encodes a transporter of cytosol glutamate into synaptic vesicles, was strongly increased in the striatum but not frontal cortex in mature GluN2D KO mice. Mice that heterozygously lack *vglut2* have been reported to show an increased locomotor response to amphetamine and increased sensitivity to the startle-disrupting effects of MK-801 [13]. However, because acute and repeated PCP administration does not increase locomotor activity in GluN2D KO mice [9], the increased expression of *vGluT2* in the striatum in GluN2D KO mice may negatively regulate PCP-induced locomotor activity. The other three genes, *Hace1*, *Smpd4*, and *Osbpl8,* have not yet been investigated with regard to motor impairment.

To investigate the role of the GluN2D subunit in PCP-induced motor impairment while avoiding the effects of GluN2D subunit ablation during development, we used the GluN2C/2D-selective antagonist UBP141 for the rotarod test. UBP141 is a competitive glutamate binding site antagonist, displaying seven-fold higher selectivity for GluN2D-containing receptors over GluN2B- or GluN2A-containing receptors [14]. In the present study, UBP141 induced severe motor impairment in WT mice but not GluN2D KO mice, suggesting that the blockade of GluN2D-containing NMDA receptors caused motor impairment without developmental adaptations. Although the affinities of UBP141 for the GluN2C and GluN2D subunits are within a similar range, UBP141 likely inhibits the GluN2C subunit as well as the GluN2D subunit. The finding that UBP141 did not induce severe motor impairment in GluN2D KO mice suggests that inhibition of GluN2C subunit-containing NMDA receptors may not be involved in motor impairment. If so, then the inhibition of GluN2C subunit-containing NMDA receptors by PCP does not appear to play a significant role in PCP-induced motor impairment. Therefore, the inhibition of the GluN2D subunit by PCP appeared to participate in PCP-induced motor impairment in WT mice.

CIQ selectively potentiates GluN2C and GluN2D subunit. The EC_50_ values for the potentiation of GluN2C- and GluN2D-containing receptors are 2.7 and 2.8 μM, respectively [15]. CIQ induced motor impairment in GluN2D KO mice but not WT mice, suggesting that CIQ-induced motor impairment is not caused by simple potentiation of the GluN2C subunit but rather by an imbalance of potentiation of the GluN2C subunit and GluN2D subunit. The GluN2C subunit mainly exists in the cerebellum, and GluN2D subunit exists in the thalamus, globus pallidus, and subthalamic nucleus. Therefore, the potentiation of the GluN2C or GluN2D subunit may affect different neural circuits.

### Induction of *c-fos* by inhibition of the GluN2D subunit or potentiation of the GluN2C subunit

UBP141 administration increased *c-fos* expression in the thalamus and striatum in WT mice but not GluN2D KO mice. Although UBP141 inhibits GluN2D and GluN2C at similar concentrations (2.2-fold selectivity) *in vitro*, UBP141 is able to inhibit GluN2C subunit-containing NMDA receptors in GluN2D KO mice. However, UBP141 did not increase *c-fos* mRNA in GluN2D KO mice (Figure 6A). In contrast, CIQ, a potentiator of GluN2C/2D subunits, induced *c-fos* expression to a similar extent in WT and KO mice (Figure 6B). Therefore, CIQ likely potentiated GluN2C subunit-containing NMDA receptors in GluN2D KO mice. These results suggest that potentiation of the GluN2C subunit and inhibition of the GluN2D subunit induce neural excitation. However, the mechanisms of *c-fos* induction by these compounds can be different. UBP-141 has a similar mechanism of action as PCP. That is, UBP-141 blocks the GluN2D subunit-containing NMDA receptor complex in the GABA neurons. The suppression of GABA neurons then disinhibits glutamatergic neurons, and activated glutamatergic neurons induce neural excitation. CIQ enhanced the function of the NMDA receptor complex by acting on GluN2C/2D subunits. In the basal ganglia and cerebellum, direct potentiation of the GluN2C/2D subunit-containing NMDA receptor complex in glutamatergic neurons may induce *c-fos* expression in the thalamus and subsequently in the striatum. Thus, these increases in *c-fos* induction by UBP141 or CIQ are likely attributable to the activation of different neural circuits.

**Figure 7:**
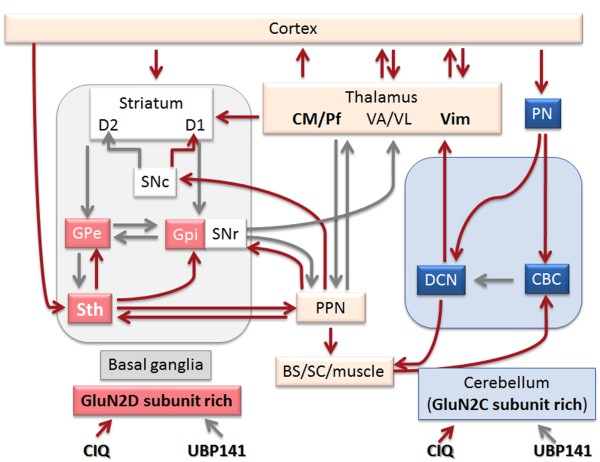
**The basal ganglia and cerebellum are involved in motor control.** Both circuitries receive projections from the cerebral cortex and send signals to the thalamus. These signals are then accumulated in the cerebral cortex. The inhibitory regulation from the basal ganglia and stimulatory regulation from the cerebellum modulate the activity of the cerebral cortex. UBP141 inhibited the NR2C subunit in both WT and GluN2D KO mice but only inhibited the NR2D subunit in WT mice. Thus, the inhibition of the GluN2D subunit is a more important factor for motor impairment than the inhibition of the GluN2C subunit. Similarly, CIQ potentiated the function of the GluN2C/2D subunit of the NMDA receptor in WT mice but potentiated only the GluN2C subunit in GluN2D KO mice. The potentiation of both the GluN2C and GluN2D subunits did not induce motor impairment in WT mice but induced motor impairment in GluN2D KO mice. Based on the balance between two competing forces, goal-directed movements could be appropriately performed. Figure is modified from [16]. Red arrows indicate excitatory pathways; gray arrows indicate inhibitory pathways. BS/SC, brain stem/spinal cord; CBC, cerebellar cortex; CM, centromedian nucleus of the thalamus; DCN, deep cerebellar nuclei; GPe, globus pallidus external segment; GPi, globus pallidus internal segment; Pf, parafascicular nucleus of the thalamus; PN, pontine nucleus; PPN, pedunculopontine nucleus; SNr, substantia nigra pars reticulate; SNc, substantia nigra pars compacta; Sth, subthalamic nucleus; VA, ventral anterior nucleus of the thalamus; Vim, ventral intermediate nucleus of the thalamus; VL, ventrolateral nucleus of the thalamus.

CIQ potentiated the function of GluN2C/2D subunit-containing NMDA receptors in WT mice but potentiated only the GluN2C subunit in GluN2D KO mice. In the present study, we found that the inhibition of GluN2D subunit-containing NMDA receptors in the motor loop or potentiation of GluN2C subunit-containing NMDA receptors likely in the cerebellum loop induced motor impairment. These two neural circuits (i.e., the basal ganglia and cerebellum) are well known to be important for cooperative movements (Figure 7). The balance between the GluN2C subunit-rich cerebellum loop and GluN2D subunit-containing basal ganglia circuit would be needed for appropriate motor performance.

### PCP-induced c-*fos* expression and basal ganglia circuitry

The immunohistochemical analysis of Fos revealed that the PCP-induced increase in the number of Fos-positive cells was suppressed in the observed brain areas in GluN2D KO mice, including the striatum, thalamus, motor cortex, and subthalamic nucleus (Figures 4 and 5). However, the numbers of Fos-positive cells in the striatum and thalamus of saline-treated GluN2D KO mice were significantly increased compared with WT mice (Figure 4(G) and Figure 5(G), respectively). There is a possibility that the existence of a ceiling effect in GluN2D KO mice might mask the PCP-induced increases in GluN2D KO mice. Nevertheless, the measurements of *c-fos* mRNA by qRT-PCR indicated no significant increase in the thalamus or striatum in GluN2D KO mice (Figure 6). Therefore, the suppression of PCP-induced *c-fos* expression in KO mice was unlikely attributable to the ceiling effect. Changes in *c-fos* mRNA are more quickly linked to neural excitation than Fos protein expression. These *c-fos* mRNA results suggested that PCP-induced excitation in the thalamus and striatum in GluN2D KO mice was suppressed as well as in other areas of the motor loop (i.e., subthalamic nucleus and motor cortex) in GluN2D KO mice. On the other hand, the observed genotypic differences in the expression of *c-fos* and activation of Fos might be secondary to PCP’s differential genotypic effects on hyperlocomotion. To delineate this issue, we used the skeletal ryanodine receptor blocker dantrolene as a muscle relaxant to relieve mice from PCP-induced hyperlocomotion. Dantrolene itself did not inhibit basal c-*fos* expression or PCP-induced c-*fos* expression in WT mice (Figure 6C, D) without PCP-induced hyperlocomotion. Thus, these results indicate that the genotypic difference in PCP-induced c-*fos* expression was not secondary to hyperlocomotion in WT mice.

These brain areas, including the striatum, thalamus, motor cortex, and subthalamic nucleus, are components of the basal ganglia circuitry that mediates various motor functions, such as movement control, behavioral performance, and motivational processes [17-21]. A standard model of the basal ganglia circuitry suggests that direct projections from the subthalamic nucleus to output structures play a major role in motor function regulation [22-24]. Interestingly, NMDA receptors in the subthalamic nucleus and globus pallidus are mainly composed of GluN1 and GluN2D subunits [25]. Therefore, genetic ablation of the GluN2D subunit likely results in the severe loss of NMDA receptor function in the subthalamic nucleus and globus pallidus in GluN2D KO mice. Acute PCP administration (2 mg/kg) increased extracellular glutamate levels in the prefrontal cortex [26]. Considering that motor and prefrontal cortical areas provide a major source of glutamatergic excitatory inputs to the subthalamic nucleus [27-29], activated glutamatergic inputs to NMDA receptors via the subthalamic nucleus would be attenuated in GluN2D KO mice. Therefore, the mechanisms responsible for resistance against PCP-induced motor impairment in GluN2D KO mice may involve dysfunction of NMDA receptors in basal ganglia circuitry caused by a lack of GluN2D subunits.

### PCP-induced changes in the expression of genes related to cellular stress are attenuated by genetic ablation of the GluN2D subunit

*Egr4*, which displays a neural-specific pattern of expression, is rapidly upregulated by neuronal activity [30-32]. Interestingly, the expression of *Egr4* (NGFI-C) was potently decreased by PCP administration in the thalamus and striatum in GluN2D KO mice (Tables 1 and 2). In contrast, *Egr4* expression in the frontal cortex in WT mice was increased by PCP administration. These results suggest that PCP administration induced differential neuronal activation in WT and GluN2D KO mice.

PCP caused selective increases in the expression of several genes in WT mice, including *Ddit4* (REDD1), *Bag-3, Txnip*, and *Sesn1*. These genes are related to cellular stress. For example, REDD1 (also known as RTP801/Dig2/DDIT4) is a critical transducer of cellular responses to energy depletion through the TSC-mTOR pathway. Mammalian REDD1 is induced following DNA damage through both p53-dependent and -independent mechanisms [33]. Recently, Jin *et al.* (2011) found that TXNIP inhibited mTOR activity by binding to and stabilizing the Redd1 protein [34]. In response to stress, p53 transcribes a group of critical negative regulators of these two pathways, including Sestrin1. BAG-3, a key component of the proteostasis machinery, is induced under oxidative stress and upon proteasome inhibition [35-37]. BAG-3 triggers the recruitment of the autophagic ubiquitin adaptor p62 and thus facilitates substrate degradation through the autophagosome lysosome pathway [35]. Therefore, the PCP-induced increases in the gene expression of *Sestrin 1*, *Bag-3*, and *Ddit4* (REDD1) in WT mice are related to cellular stress, DNA damage, and the autophagosome lysosome pathway. The lack of increased expression of these genes in GluN2D KO mice after PCP administration suggests that ablation of the GluN2D subunit may prevent PCP-induced cellular stress in the striatum and frontal cortex.

### Concluding remarks

In the present study, we found that PCP-induced motor impairment was reduced in GluN2D KO mice. Although the GluN2D subunit population is rather low and restricted in the mature brain, we conclude that the GluN2D subunit plays an important role in PCP-induced motor impairment and gene expression.

## Materials and methods

### Materials

Phencyclidine (Shionogi Pharmaceutical Co. Ltd., Osaka, Japan) was dissolved in saline. UBP141 (Abcam Biochemicals; http://www.ascentscientific.com/; accessed June 7, 2013) was dissolved in twice an equal molar amount of NaOH solution, diluted with saline, and neutralized to pH 8.0. Prof. Stephen F. Traynelis kindly provided CIQ to us. CIQ was dissolved in dimethylacetamide, and four volumes of PEG-400 were then added and gently mixed. Finally, five volumes of 5% glucose solution were added. After preparation, the CIQ solution was immediately used. Dantrolene (Sigma-Aldrich, St. Louis, MO, USA) was freshly dissolved in warm water (50°C) by sonication.

### Animals

The experimental protocols were approved by the Animal Use and Care Committee of the Tokyo Metropolitan Institute of Medical Science. The mice were housed 5–6 per cage with free access to food and water and maintained on a 12 h/12 h light/dark cycle.

Three-month-old homozygous GluN2D mutant and WT mice of both sexes were obtained by crossing F11 heterozygous GluN2D mutant mice (+/−) on a 99.99% pure C57BL/6 genetic background [2]. WT and homozygous KO mice were genotyped by polymerase chain reaction (PCR) using two internal primers (one targeted at the neomycin-resistant gene [NEO] insertion in the KO construct and one targeted at the WT gene) and one external primer, generating two products that identified the WT and KO genes. Polymerase chain reaction using Go Taq DNA polymerase (Promega, Madison, WI, USA) was performed on ear DNA eluted after overnight digestion by proteinase K. The forward primer (5′-GAG ATT GAG ATG CTG GAG CGG CTG-3′) and WT primer (5′-CGG CGG TGG CGG GGG TTT GGC-3′) produced a 455-base pair (bp) band, whereas the forward primer and NEO primer (5′-GTG GAT GTG GAA TGT GTG CGA GGC-3′) produced a 184-bp band. The PCR amplification products were electrophoretically separated on 2% agarose gels, and the bands were visualized under ultraviolet illumination.

### Rotarod test

A commercially available rotarod apparatus was used (KN-75, Natsume Seisakusho Co., Ltd, Tokyo, Japan). During the training phase, the mice underwent a training session on the apparatus for five training trials, in which the rod (3 cm diameter) was maintained at a constant speed for 300 s. The rod was kept stationary for the first trial and held at 6 rotations per minute (rpm) for two trials. The rod was then rotated at 12 rpm for two training trials, with a 3-h intertrial interval (ITI). The mice that were able to stay on the rod at 12 rpm for more than 200 s were evaluated in the test phase the next day (day 2 in Figure 1A). After the training phase, 93.3% and 90.9% of the WT and GluN2D KO mice, respectively, had proceeded to the test phase.

During the test phase, two trials were conducted with a 3-h ITI. In the first trial, the mice were placed on the rod that rotated at 12 rpm, at which time a timer was started and maintained for 300 s (day 3 in Figure 1A). The second trial was performed 30 min after PCP (3 mg/kg, subcutaneously [s.c.]) and CIQ (20 mg/kg, intraperitoneally [i.p.]) administration at 12 rpm or UBP141 administration (24.3 μg, 20 μl of 3 mM per mouse, intracisternally [i.c.], under diethyl ether anesthesia) at 0 rpm. The second trial lasted 300 s. The latency to fall from the rod was recorded for each of the two successive trials. For the animal studies, Fisher’s exact test was used to compare the data.

### Tissue preparation, RNA isolation, probe labeling, and microarray hybridization

#### Preliminary experiment: Gene expression analyses using cDNA array

WT and GluN2D KO mice were injected with saline or PCP (10 mg/kg, s.c.). Two hours after the injection, the striata from 10 mice per treatment group (a total of 160 mice were used) were quickly dissected on ice and immediately frozen at −80°C. The precise procedure for the cDNA array experiments is described elsewhere [38]. This developed a cDNA array system that utilized mouse KIAA-homologous cDNA (mKIAA) clones (http://www.kazusa.or.jp/rouge/; accessed July 7, 2013). KIAA clones were originally obtained from a human cDNA sequencing project to accumulate protein-coding sequence information on unidentified human genes. Although unidentified mKIAA genes are interesting, the expression data of newly identified mKIAA genes is not suitable for pathway analysis.

#### Experiment 1. Gene expression analyses using Illumina DNA array

WT and GluN2D KO mice were injected with saline or PCP (10 mg/kg, s.c.). Four groups, consisting of saline-treated WT, saline-treated GluN2D KO, PCP-treated WT, and PCP-treated GluN2D KO mice, were prepared. One group included four samples. One sample is prepared with more than three mice. We used 16 samples and the16 cDNA arrays. Total RNA was isolated with TRIzol reagent (Invitrogen, Carlsbad, CA, USA) and purified using the RNeasy Mini Kit according to the manufacturer’s instructions (Qiagen, Valencia, CA, USA). RNA was quantified by measuring the optical density at 260 nm using a spectrophotometer (Beckman, Fullerton, CA, USA).

Gene expression profiling for 16 different RNA samples was performed using microarray platforms from Illumina (San Diego, CA, USA). For the Illumina platform, we used the multi-sample MouseRef-8 BeadChip format with greater than 24,000 probes that simultaneously profile eight samples on a single chip. Briefly, 500 ng of total RNA was labeled using an Illumina TotalPrep RNA Amplification Kit (Life Technologies, Carlsbad, CA, USA). Double-stranded cDNA was synthesized using T7-oligo (dT) primers followed by an *in vitro* transcription reaction to amplify antisense-RNA (aRNA). Biotin was incorporated into the synthesized aRNA target. The biotinylated cRNA target was hybridized to the MouseRef-8 BeadChip. Hybridization, washing, and scanning were performed according to the manufacturer’s instructions following overnight hybridization. The chips were scanned using a BeadScan (Illumina) at a multiplier setting of “2.” The microarray images were recorded, and gene expression data were automatically extracted according to the manufacturer’s default settings. Raw microarray intensity data were provided using differential expression algorithms (Illumina). Additional calculations were performed within a Microsoft Excel spreadsheet. For each probe, intensity data were used in *t*-tests to obtain *p*-values and fold changes. Genes with a fold change > 1.3 or < 0.7 and between-group differences with *p* < 0.05 were considered to be differentially expressed genes (DEGs; [39]. The pathway analyses of statistically significant genes were performed using MetaCore software (GeneGo, St. Joseph, MI, USA). The figures in the text are adaptations based on the figures created by MetaCore software.

### Real-time qRT-PCR

To confirm the cDNA array results, quantitative reverse-transcription PCR (qRT-PCR) was performed. Purified mRNA (0.5 μg) was reverse-transcribed with random nonamers using the SuperScript III first-strand synthesis system according to the manufacturer’s instructions (Invitrogen). To evaluate gene expression, real-time qRT-PCR was performed using the SYBR-green-based real-time qRT-PCR assay. Using the primers identified by Lasergene (DNASTAR, Madison, WI, USA), qRT-PCR was performed. First-strand cDNA from the pooled mRNA was used as a template in a 25-μl PCR reaction with 200 nM primers and the Power SYBR Green PCR Master Mix (Applied Biosystems, Warrington, UK). Forty-five cycles of PCR were performed with an Applied Biosystems 7300 Real Time PCR System (Applied Biosystems). The levels of all of the cDNAs generated from the mRNA by reverse transcription were calculated using the standard curve method for quantification and normalized to actin transcript levels.

To confirm the Illumina DNA array results, qRT-PCR experiments were performed using LightCycler 480 and LightCycler 480 Probes Master (Roche, Indianapolis, IN, USA) according to the manufacturer’s protocol. Sequences for gene-specific primers that corresponded to the PCR targets were obtained using LightCycler Probe Design software (Roche). The primers were synthesized and purified by high-performance liquid chromatography by Nihon Gene Research Laboratories (Sendai, Japan). Quantitative PCR values were normalized to actin levels.

To examine c-*fos* mRNA levels by qRT-PCR, the mice were injected with UBP141, CIQ, and control vehicles similarly to the procedure used in the rotarod test but without behavioral testing. Two hours after injection, the thalamus and striatum from each mouse were quickly dissected on ice and immediately frozen at −80°C. Similarly, the mice first received dantrolene (5 mg/kg, i.p.) and then injected with PCP (10 mg/kg, s.c.) 15 min later. Two hours after the PCP injection, the thalamus and striatum from each mouse were quickly dissected on ice and immediately frozen at −80°C. Total RNA was separately extracted from each tissue. The qRT-PCR experiments were performed using LightCycler 480 and LightCycler 480 Probes Master (Roche, Indianapolis, IN, USA) according to the manufacturer’s protocol.

### Immunohistochemistry

All of the animals were deeply anesthetized with an overdose of sodium pentobarbital (50 mg/kg, i.p.; Nembutal, Dainippon-Sumitomo, Osaka, Japan) and transcardially perfused with 50 ml phosphate-buffered saline (PBS), followed by ice-cold 4% paraformaldehyde in 0.1 M phosphate buffer (PB; pH 7.4). The brains were quickly removed from the skull and post-fixed overnight in the same fixative. After post-fixation, the brains were cryoprotected in 20% sucrose in PB (0.1 M, pH 7.4) for 2 days at 4°C and serially sectioned (50-μM thickness) using a Leica VT1000 P vibratome (Leica Microsystems). For the immunohistochemical visualization of Fos expression, coronal brain sections were pretreated for 15 min at room temperature with a 1% hydrogen peroxide solution to remove peroxidases. The sections were blocked for 2 h at room temperature in 4% goat serum solution (0.05% Tween-20/Tris-buffered saline), followed by incubation for 48 h at 4ºC in the primary Fos antibody (anti-Fos; 1:10,000; Ab-5, Calbiochem) in PBS with 4% normal goat serum. Immunoreactivity was visualized using ImmPRESS reagent (Vector Labs, Burlingame, CA, USA) with 3,3′-diaminobenzidine/nickel as the chromogen. Control and experimental tissues from each group were processed in parallel. No staining was observed in the brain sections with omission of either the primary or secondary antibody.

### Cell counting

The number of Fos-positive neurons within the confines of each anatomically demarcated nucleus was evaluated using bright-field microscopy at 20× magnification. Based on Fos expression and its relevance to motor function, the following brain regions were selected for counting Fos-immunoreactive cells: striatum (0.98 mm), thalamus (−2.06 mm), paraventricular thalamic nucleus (−1.46), cingulate cortex and motor cortex (1.7 mm), and subthalamic nucleus (−2.18 mm). Bregma-based coordinates [10] are shown in parentheses after each brain region. The analyses of Fos-immunoreactive nuclei were performed using a Keyence microscope system (Keyence BZ-8100, Woodcliff Lake, NJ, USA). Twenty to 30 photographs (one photograph was extracted from five superimposed photographs at different depths) per coronal slice were taken in the selected regions. Full-image reconstruction of the entire slice was performed using Keyence BZ-8100. The areas that contained the striatum and other brain areas were cut off using Adobe Photoshop Elements software and transferred to NIH ImageJ software to automatically count Fos-labeled cells in the region of interest as defined by the template. The number of Fos-positive cells was normalized to the area (mm^2^).

### Statistical analyses

Parametric or nonparametric analysis of variance (ANOVA) and appropriate *post hoc* tests were used to assess the effects of treatments, depending on the initial analyses of distribution normality. Correlation analyses were performed using Pearson’s coefficient. In the rotarod test, the data after 300 s were not recorded because the measurement of motor performance concluded at 300 s. To analyze the results from the rotarod test, Fisher’s exact test was used. Statistical significance was set at *p* < 0.05.

## Competing interests

The authors declare no competing financial interests.

## Authors’ contributions

HY designed the research; HY, EK, WS, RH, TY, YH, and YT performed the research; HY, EK, WS, RH, TY, HK, and KI analyzed the data; HY, TY, HK, MM, and KI prepared the manuscript. All authors read and approved the final manuscript.

## Supplementary Material

Additional file 1: Table S1 **Calculated gene expression data from raw microarray intensity data (multi-sample MouseRef-8 BeadChip).** WPCP, PCP-injected WT mice; WS, saline-injected WT mice; KOS, saline-injected GluN2D KO mice; KOPCP, PCP-injected GluN2D KO mice. Click here for file

Additional file 2: Table S2 **Numerical data of Figure **6**(*c-fos/actin*) and these reference data (*actin*).** Two hours after the injection of **(A)** UBP141 (3 mM, 20 μl, i.c.), **(B)** CIQ (20 mg/kg, i.p.), **(C, D)** Dantrolene (5 mg/kg, i.p.) and PCP (10 mg/kg, s.c), c-*fos* and *actin* expressions were measured using qRT-PCR analyses. The ratio of c-*fos*/*actin* transcripts and its reference *actin* transcripts are shown as the mean ± SEM fold change from saline controls in WT mice. Click here for file

## References

[B1] Watanabe M Inoue Y Sakimura K Mishina M Developmental changes in distribution of NMDA receptor channel subunit mRNAs Neuroreport 1992 6 1138 1140 10.1097/00001756-199212000-00027 1493227

[B2] Ikeda K Araki K Takayama C Inoue Y Yagi T Aizawa S Mishina M Reduced spontaneous activity of mice defective in the epsilon 4 subunit of the NMDA receptor channel Brain Res Mol Brain Res 1995 6 61 71 10.1016/0169-328X(95)00107-4 8774946

[B3] Miyamoto Y Yamada K Noda Y Mori H Mishina M Nabeshima T Lower sensitivity to stress and altered monoaminergic neuronal function in mice lacking the NMDA receptor epsilon 4 subunit J Neurosci 2002 6 2335 2342 11896172 10.1523/JNEUROSCI.22-06-02335.2002 PMC6758257

[B4] Selemon LD Goldman-Rakic PS The reduced neuropil hypothesis: a circuit based model of schizophrenia Biol Psychiatry 1999 6 17 25 10.1016/S0006-3223(98)00281-9 9894571

[B5] Krystal JH D’Souza DC Mathalon D Perry E Belger A Hoffman R NMDA receptor antagonist effects, cortical glutamatergic function, and schizophrenia: toward a paradigm shift in medication development Psychopharmacology (Berl) 2003 6 215 233 10.1007/s00213-003-1582-z 12955285

[B6] Geyer M Krebs-Thomson K Braff D Swerdlow N Pharmacological studies of prepulse inhibition models of sensorimotor gating deficits in schizophrenia: a decade in review Psychopharmacology (Berlin) 2001 6 117 154 10.1007/s002130100811 11549216

[B7] Carlsson M Carlsson A The NMDA antagonist MK-801 causes marked locomotor stimulation in monoamine-depleted mice J Neural Transm 1989 6 221 226 10.1007/BF01258633 2538557

[B8] Lindahl JS Keifer J Glutamate receptor subunits are altered in forebrain and cerebellum in rats chronically exposed to the NMDA receptor antagonist phencyclidine Neuropsychopharmacology 2004 6 2065 2073 10.1038/sj.npp.1300485 15138442

[B9] Hagino Y Kasai S Han W Yamamoto H Nabeshima T Mishina M Ikeda K Essential role of NMDA receptor channel epsilon4 subunit (GluN2D) in the effects of phencyclidine, but not methamphetamine PLoS One 2010 6 e13722 10.1371/journal.pone.0013722 21060893 PMC2965660

[B10] Franklin K Paxinos G The Mouse Brain in Stereotaxic Coordinates 2007 3 Amsterdam: Academic Press/Elsevier

[B11] Kobayashi S Yano M Suetomi T Ono M Tateishi H Mochizuki M Xu X Uchinoumi H Okuda S Yamamoto T Dantrolene, a therapeutic agent for malignant hyperthermia, markedly improves the function of failing cardiomyocytes by stabilizing interdomain interactions within the ryanodine receptor J Am Coll Cardiol 2009 6 1993 2005 10.1016/j.jacc.2009.01.065 19460614 PMC2764410

[B12] Traynelis SF Wollmuth LP McBain CJ Menniti FS Vance KM Ogden KK Hansen KB Yuan H Myers SJ Dingledine R Glutamate receptor ion channels: structure, regulation, and function Pharmacol Rev 2010 6 405 496 10.1124/pr.109.002451 20716669 PMC2964903

[B13] Naert A Callaerts-Vegh Z Moechars D Meert T D’Hooge R Vglut2 haploinsufficiency enhances behavioral sensitivity to MK-801 and amphetamine in mice Progress in Neuro-Psychopharmacology and Biological Psychiatry 2011 6 1316 1321 10.1016/j.pnpbp.2011.03.023 21514350

[B14] Costa B Feng B Tsintsadze T Morley R Irvine M Tsintsadze V Lozovaya N Jane D Monaghan D N-methyl-D-aspartate (NMDA) receptor NR2 subunit selectivity of a series of novel piperazine-2,3-dicarboxylate derivatives: preferential blockade of extrasynaptic NMDA receptors in the rat hippocampal CA3-CA1 synapse J Pharmacol Exp Ther 2009 6 618 626 10.1124/jpet.109.156752 19684252 PMC2775268

[B15] Mullasseril P Hansen KB Vance KM Ogden KK Yuan H Kurtkaya NL Santangelo R Orr AG Le P Vellano KM Liotta DC Traynelis SF A subunit-selective potentiator of NR2C- and NR2D-containing NMDA receptors Nat Commun 2010 6 90 20981015 10.1038/ncomms1085 PMC3113701

[B16] Kringelbach ML Jenkinson N Owen SL Aziz TZ Translational principles of deep brain stimulation Nat Rev Neurosci 2007 6 623 635 10.1038/nrn2196 17637800

[B17] Hamada I DeLong MR Excitotoxic acid lesions of the primate subthalamic nucleus result in transient dyskinesias of the contralateral limbs J Neurophysiol 1992 6 1850 1858 1479448 10.1152/jn.1992.68.5.1850

[B18] Baunez C Nieoullon A Amalric M In a rat model of parkinsonism, lesions of the subthalamic nucleus reverse increases of reaction time but induce a dramatic premature responding deficit J Neurosci 1995 6 6531 6541 7472415 10.1523/JNEUROSCI.15-10-06531.1995 PMC6578020

[B19] Baunez C Amalric M Robbins TW Enhanced food-related motivation after bilateral lesions of the subthalamic nucleus J Neurosci 2002 6 562 568 11784803 10.1523/JNEUROSCI.22-02-00562.2002 PMC6758660

[B20] Baunez C Robbins TW Bilateral lesions of the subthalamic nucleus induce multiple deficits in an attentional task in rats Eur J Neurosci 1997 6 2086 2099 10.1111/j.1460-9568.1997.tb01376.x 9421169

[B21] Dybdal D Gale K Postural and anticonvulsant effects of inhibition of the rat subthalamic nucleus J Neurosci 2000 6 6728 6733 10964979 10.1523/JNEUROSCI.20-17-06728.2000 PMC6772946

[B22] Alexander GE Crutcher MD Functional architecture of basal ganglia circuits: neural substrates of parallel processing Trends Neurosci 1990 6 266 271 10.1016/0166-2236(90)90107-L 1695401

[B23] DeLong MR Primate models of movement disorders of basal ganglia origin Trends Neurosci 1990 6 281 285 10.1016/0166-2236(90)90110-V 1695404

[B24] Parent A Hazrati LN Functional anatomy of the basal ganglia. II. The place of subthalamic nucleus and external pallidum in basal ganglia circuitry Brain Res Brain Res Rev 1995 6 128 154 10.1016/0165-0173(94)00008-D 7711765

[B25] Standaert DG Testa CM Young AB Penney JB Jr Organization of N-methyl-D-aspartate glutamate receptor gene expression in the basal ganglia of the rat J Comp Neurol 1994 6 1 16 10.1002/cne.903430102 8027428

[B26] Amitai N Kuczenski R Behrens MM Markou A Repeated phencyclidine administration alters glutamate release and decreases GABA markers in the prefrontal cortex of rats Neuropharmacology 2012 6 1422 1431 10.1016/j.neuropharm.2011.01.008 21238466 PMC3107933

[B27] Afsharpour S Topographical projections of the cerebral cortex to the subthalamic nucleus J Comp Neurol 1985 6 14 28 10.1002/cne.902360103 2414329

[B28] Canteras NS Shammah-Lagnado SJ Silva BA Ricardo JA Afferent connections of the subthalamic nucleus: a combined retrograde and anterograde horseradish peroxidase study in the rat Brain Res 1990 6 43 59 10.1016/0006-8993(90)91087-W 2350684

[B29] Bevan MD Francis CM Bolam JP The glutamate-enriched cortical and thalamic input to neurons in the subthalamic nucleus of the rat: convergence with GABA-positive terminals J Comp Neurol 1995 6 491 511 10.1002/cne.903610312 8550895

[B30] Crosby S Puetz J Simburger K Fahrner T Milbrandt J The early response gene NGFI-C encodes a zinc finger transcriptional activator and is a member of the GCGGGGGCG (GSG) element-binding protein family Mol Cell Biol 1991 6 3835 3841 2072895 10.1128/mcb.11.8.3835-3841.1991 PMC361165

[B31] Crosby S Veile R Donis-Keller H Baraban J Bhat R Simburger K Milbrandt J Neural-specific expression, genomic structure, and chromosomal localization of the gene encoding the zinc-finger transcription factor NGFI-C Proc Natl Acad Sci U S A 1992 6 4739 4743 10.1073/pnas.89.10.4739 1584812 PMC49159

[B32] Honkaniemi J Sharp F Prolonged expression of zinc finger immediate-early gene mRNAs and decreased protein synthesis following kainic acid induced seizures Eur J Neurosci 1999 6 10 17 10.1046/j.1460-9568.1999.00401.x 9987007

[B33] Ellisen LW Ramsayer KD Johannessen CM Yang A Beppu H Minda K Oliner JD McKeon F Haber DA REDD1, a developmentally regulated transcriptional target of p63 and p53, links p63 to regulation of reactive oxygen species Mol Cell 2002 6 995 1005 10.1016/S1097-2765(02)00706-2 12453409

[B34] Jin HO Seo SK Kim YS Woo SH Lee KH Yi JY Lee SJ Choe TB Lee JH An S TXNIP potentiates Redd1-induced mTOR suppression through stabilization of Redd1 Oncogene 2011 6 3792 3801 10.1038/onc.2011.102 21460850

[B35] Arndt V Dick N Tawo R Dreiseidler M Wenzel D Hesse M Furst DO Saftig P Saint R Fleischmann BK Chaperone-assisted selective autophagy is essential for muscle maintenance Curr Biol 2010 6 143 148 10.1016/j.cub.2009.11.022 20060297

[B36] Dikshit P Jana NR The co-chaperone CHIP is induced in various stresses and confers protection to cells Biochem Biophys Res Commun 2007 6 761 765 10.1016/j.bbrc.2007.04.018 17442270

[B37] Jacobs AT Marnett LJ HSF1-mediated BAG3 expression attenuates apoptosis in 4-hydroxynonenal-treated colon cancer cells via stabilization of anti-apoptotic Bcl-2 proteins J Biol Chem 2009 6 9176 9183 10.1074/jbc.M808656200 19179333 PMC2666569

[B38] Yamamoto H Imai K Takamatsu Y Kamegaya E Kishida M Hagino Y Hara Y Shimada K Yamamoto T Sora I Methamphetamine modulation of gene expression in the brain: analysis using customized cDNA microarray system with the mouse homologues of KIAA genes Brain Res Mol Brain Res 2005 6 40 46 10.1016/j.molbrainres.2005.02.028 15950759

[B39] Liao G Wen Z Irizarry K Huang Y Mitsouras K Darmani M Leon T Shi L Bi X Abnormal gene expression in cerebellum of Npc1−/− mice during postnatal development Brain Res 2010 6 128 140 20153740 10.1016/j.brainres.2010.02.019 PMC2848886

